# Severity of COVID-19 and diabetes mellitus: there is still a lot to be learned

**DOI:** 10.20945/2359-3997000000261

**Published:** 2020-06-05

**Authors:** Melanie Rodacki

**Affiliations:** 1 Universidade Federal do Rio de Janeiro Rio de Janeiro RJ Brasil Universidade Federal do Rio de Janeiro, Rio de Janeiro, RJ, Brasil

In this issue of *Archives of Endocrinology and Metabolism* , Pinto and cols. have performed a meta-analysis to investigate the association between type 2 diabetes (T2D) and the severity of COVID-19. The outcomes show that T2D seems to be a major, age-independent risk factor for the severity of this infection. The authors have included the first 7 published articles with a total of 1592 subjects, with data collected exclusively in the Chinese population, where the pandemic started (1). Simultaneously, other meta-analysis with slightly different search terms have also been published and indicated the same association (2,3,4). Although one of these publications included not only Chinese subjects (2), they still represent the vast majority of the studied cases. Therefore, it is crucial to obtain data from other populations where COVID-19 emerged most recently. Small studies in non-Chinese populations have recently became available and suggest that diabetes represent an important risk factor for COVID-19 severity not only in China, but also worldwide (5,6).

Diabetes has also been linked to an increased severity of bacterial and other viral infections, including H1N1 influenza (7,8). However, COVID-19 has been associated predominantly with T2D and, at this point, there are no reports of increased frequency of severe COVID-19 cases in children, adolescents and young patients with type 1 diabetes (T1D). Further studies are still required to clarify if subjects with T1D and T2D really have a difference in the severity of COVID-19 or if this is a reflection of the larger proportion of T2D cases in comparison to T1D. One could suggest that the potential differentiation in risk for T1D and T2D might be explained exclusively by the age of the affected patients, which is generally younger in subjects with T1D. Interestingly, Pinto and cols. did not observe an impact of the patients’ age in the association between diabetes and the severity of COVID-19 in this population exclusively with T2D. This suggests that other factors, such as obesity, comorbidities, metabolic syndrome and a chronic subclinical inflammatory state might be responsible for a preferential association between COVID-19 and T2D rather than T1D (9), although it is still important to further investigate the association of age and severity of COVID-19 in subjects with both types of diabetes and a broader age range. Obesity has recently been recognized as an important independent risk factor for the severity of COVID-19 (10), which reinforces this possibility. The potential role of obesity and metabolic syndrome in the association between diabetes and the severity of COVID-19 could raise the question if hyperglycemia itself would have any influence in the outcomes of patients with COVID-19. The evidence points out that hyperglycemia worsens the prognosis and increases the risk of death, especially hyperglycemia at hospital admission (11-13). Acute hyperglycemia increases the production of inflammatory mediators that could potentialize the “cytokines storm” observed in severe COVID-19 cases (14). Although novel information is daily added to the current knowledge of the association between diabetes and COVID-19, there is still a long pathway to be unveiled in order to decrease the frequency of severe COVID-19 and death in subjects with diabetes (especially in T2D), until the vaccine and treatment options become available for clinical use.
